# Macrophage-related immune responses to polyetherketoneketone bone implants: Single-cell transcriptome analysis

**DOI:** 10.1016/j.mtbio.2025.102257

**Published:** 2025-08-28

**Authors:** Jiannan Zhou, Huibin Liang, Jiahao Chen, An Li, Jingtao Dai, Ping Li

**Affiliations:** a School and Hospital of Stomatology, Guangdong Engineering Research Center of Oral Restoration and Reconstruction & Guangzhou Key Laboratory of Basic and Applied Research of Oral Regenerative Medicine, Guangzhou Medical University, Guangzhou, China; b Department of Prosthodontics, Geriatric Dentistry and Craniomandibular Disorders, Charité-Universitätsmedizin Berlin, Corporate Member of Freie Universität Berlin and Humboldt-Universität zu Berlin, Berlin, Germany; c Department of Periodontology, Stomatological Hospital, School of Stomatology, Southern Medical University, Guangzhou, China; d Department of Orthodontics, Stomatological Hospital, School of Stomatology, Southern Medical University, Guangzhou, China

**Keywords:** Macrophage, Bone, Polyetherketoneketone, Titanium, RNA sequencing

## Abstract

Polyetherketoneketone (PEKK) has emerged as a potential alternative to titanium (Ti) for bone implants. Nevertheless, its osseointegration performance is inferior to that of Ti, primarily due to the limited understanding of its early immune reactions. To address this limitation, this study utilized single-cell RNA sequencing to investigate the distinct early macrophage responses triggered by Ti-based and PEKK-based implants. This approach enabled the characterization of macrophage-polarization dynamics and intercellular interactions within the bone-marrow microenvironment post-implantation. The findings revealed a material-dependent dichotomy in macrophage phenotype: Ti implants preferentially recruited *Cd99*^+^ macrophages, establishing an anti-inflammatory microenvironment that promotes osseointegration. Conversely, PEKK implants recruited *Icam1*^+^ macrophages, leading to persistent inflammation and hematopoietic stem cells (HSCs) stress. Additionally, Ti surfaces facilitated CD99-dependent crosstalk between macrophages and T cells, enhancing Th2 responses, which are indicative of an anti-inflammatory effect. In contrast, PEKK-associated macrophages triggered ICAM1-driven necroptosis in HSCs, disrupting hematopoietic homeostasis. These results indicate the early macrophage-related responses as key determinants of the clinical-outcome differences between Ti and PEKK implants.

## Highlights

• Titanium and polyetherketoneketone (PEKK) evoke distinct macrophage responses.
• Titanium promotes regeneration via the anti-inflammatory *Cd99*^+^ macrophages.
• PEKK-caused inflammation via *Icam1*^+^ macrophages perturbs hematopoietic stem cells.
• Single-cell RNA sequencing advances biomaterial immunogenicity evaluation.

## Graphical abstract

**Figure:**
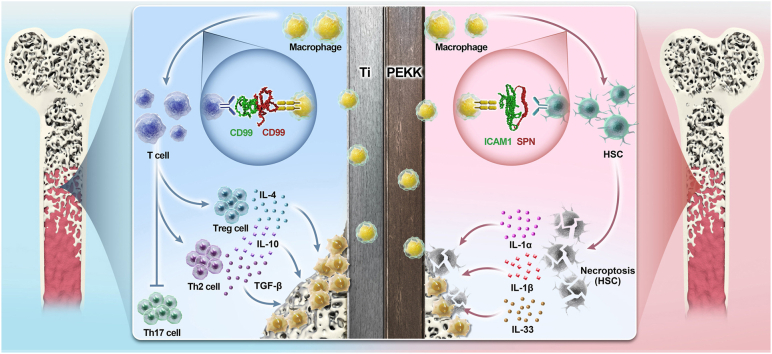

## 1 Introduction

Polyetheretherketone (PEEK), a high-performance thermoplastic, has gained prominence in orthopedic and trauma surgery due to its biocompatibility, mechanical strength, and radiolucency [1,2]. Among PEEK variants, polyetherketoneketone (PEKK) has broad clinical applications, including spinal stabilization, complex fracture fixation, arthroplasty components, and cranioplasty [[3], [4], [5]]. Experimental studies have demonstrated that PEKK scaffolds promote osteogenic differentiation in animal models, with micro–computed tomography, histological, and mechanical analyses revealing significantly superior bone growth with PEKK compared to PEEK. The volume of newly formed bone was significantly increased that observed with PEKK [6]. The enhanced protein-adsorption kinetics of PEKK facilitate vascular infiltration and osteoprogenitor-cell recruitment, highlighting its excellent biocompatibility [7]. However, previous studies have demonstrated that PEKK implants exhibit inferior osseointegration compared to titanium (Ti)-based implants, which may limit their clinical applications [8,9]. This discrepancy arises from inherent differences in biomaterial-biological interactions, particularly variations in surface characteristics and immune responses [10,11].

Biological responses to implanted biomaterials are primarily mediated by activation of the immune system [12,13]. Upon implantation, protein adsorption on the material surface triggers a cascade of cellular events, beginning with neutrophil recruitment, followed by macrophage infiltration, and ultimately engaging both innate and adaptive immune responses [14,15]. This sequential progression of immune cells plays a decisive role in directing tissue integration and determining the long-term functionality of the implant [15,16]. Notably, emerging evidence indicates that the macrophage response elicited by biomaterial surfaces can profoundly shape downstream biological processes and outcomes [17,18]. Macrophages perform essential functions, including (1) phagocytosis of debris and pathogens during early inflammation, (2) secretion of growth factors such as vascular endothelial growth factor and platelet-derived growth factor to promote angiogenesis and fibroblast activation, and (3) facilitation of tissue repair through M2 polarization, which promotes remodeling of the extracellular matrix [19,20]. However, the macrophage-related response to PEKK-based implants remains poorly characterized, particularly during the early immune response, when surface-activated macrophages significantly influence subsequent regeneration.

Single-cell transcriptomics investigates complex biological processes with unprecedented resolution and depth [21,22]. Regarding immunology, this technology offers a promising approach to unravel the mechanisms underlying immune regulation and tissue regeneration [23,24]. Applying single-cell RNA sequencing (scRNA-seq) to biomaterial–tissue interactions has yielded key insights into the cellular heterogeneity and molecular pathways that shape host responses to implanted materials [25,26]. This approach has enabled the identification of discrete immune and stromal cell populations involved in the foreign body response. For instance, Jia Li et al. have utilized scRNA-seq to map the osteoimmune landscape in murine models, revealing a pivotal intercellular communication network between neutrophils and hematopoietic stem cells (HSCs), mediated by the CXCL12/CXCR3 signaling axis [27]. Despite such progress, the osteoimmune microenvironment associated with PEKK-based implants remains obscure.

**Scheme 1:**
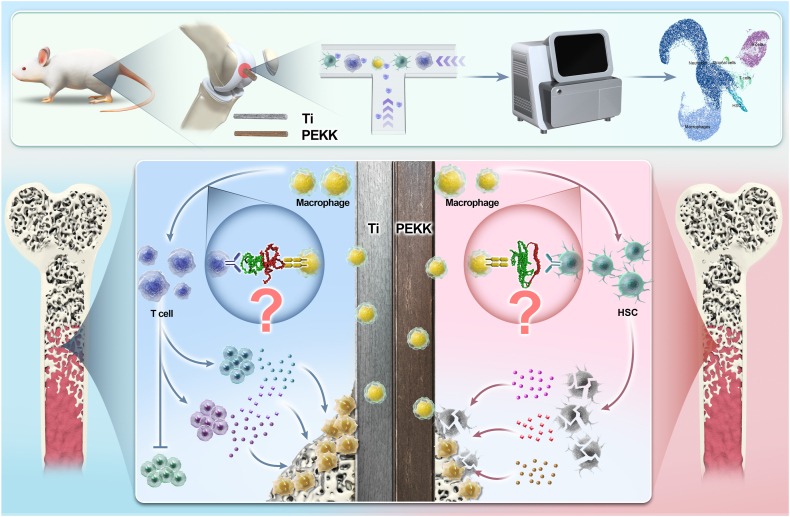
Schematic illustration of macrophage-related immune responses to titanium versus polyetherketoneketone implants in the bone microenvironment, determined by single-cell RNA sequencing.

This study aimed to elucidate the mechanisms driving the early macrophage response to PEKK-based versus Ti-based implants. To this end, scRNA-seq was employed to generate a high-resolution atlas of the cellular response, enabling detailed characterization of immune-cell dynamics and functional states. The analytical framework focused on the following three main objectives: (1) to define immune subset-specific molecular signatures elicited by PEKK-based versus Ti-based implants, with an emphasis on differential gene expression; (2) to model intercellular communication via surface-receptor-ligand interactions and validate spatially resolved signaling; and (3) to link biomaterial-specific cues to cellular outcomes through pathway enrichment analysis and experiments (Scheme 1).

## 2 Materials and methods

### 2.1 Preparation of materials

Medical certified Ti-based alloys (Ti-6Al-4V, Titanium Grade 5, Foshan Angels Biotechnology Co.,Ltd.) were fabricated into circular discs (10 mm × 10 mm × 1 mm) for *in vitro* assays and cylindrical rods (1 mm × 1 mm × 1.5 cm) for *in vivo* studies. The substrates were sequentially polished using silicon carbide abrasive papers with grit sizes ranging from 400 to 2000 to ensure surface uniformity. They were then ultrasonically cleaned in acetone, 75 % ethanol, and deionized water for 20 min each. Finally, the samples underwent autoclave sterilization, drying, and ultraviolet irradiation for 3 h before experimentation.

Medical-grade PEKK (SEQENS, Hangzhou Lexsyn New Materials Technology Co., Ltd) was fabricated into circular discs (10 mm × 10 mm × 1 mm) for *in vitro* assays and cylindrical rods (1 mm × 1 mm × 1.5 cm) for *in vivo* studies. PEKK granules were vacuum-dried at 120 °C for 4 h to eliminate moisture, then processed via injection molding at 380 °C with a mold temperature of 160 °C. After pressure holding and cooling, the molded samples were demolded. Surface finishing was performed using silicon carbide abrasive papers (grit sizes 600, 800, and 1200), followed by polishing with ultrafine microfiber cloths soaked in anhydrous ethanol to enhance smoothness. The samples were then ultrasonically cleaned in 75 % ethanol, and deionized water for 15 min each, then dried at 60 °C for 12 h. Finally, low-temperature plasma sterilization was performed for 2 h.

### 2.2 Surface characterization

The surface morphologies of the Ti and PEKK specimens were characterized using a field emission scanning electron microscope (FE-SEM, JSM-7001F, JEOL, Japan) at an accelerating voltage of 10 kV. Elemental distribution on the sample surfaces was examined using energy-dispersive X-ray spectroscopy (EDS, QX200, Bruker Optics, Germany). Surface roughness was measured using a non-contact confocal laser scanning microscope (VK-X3000 series, KEYENCE, Germany) at × 20 magnification. Surface wettability was evaluated using a contact-angle analyzer (JC2000DF, Powereach, China) via the static-drop method. Five parallel samples and three fields of view per sample were analyzed.

### 2.3 Cell culture

The mouse macrophage cell line RAW 264.7 (Procell Life Science and Technology Co., Ltd., Wuhan, China) was cultured under standard conditions (37 °C, 5 % CO_2_) [28]. For experiments, cells from passages 4–7 were seeded into 6-well plates at a density of 5 × 10^5^ cells per well. All the *in vitro* assays were performed in triplicate across three independent experiments to ensure statistical reliability.

### 2.4 Protein adsorption

Ti and PEKK specimens (n = 3) were incubated in a 5 mg/mL bovine serum albumin (BSA) solution in phosphate-buffered saline (PBS) at 37 °C for 4 h. Afterward, they were gently rinsed with PBS to remove any residual unbound proteins. The concentration of adsorbed protein was measured using a microplate reader at 525 nm. A standard curve was generated using the BCA protein assay kit (P0011, Beyotime, China) and the protein adsorption levels on Ti and PEKK surfaces were determined accordingly.

### 2.5 Calcein-acetoxymethyl (AM)/propidium iodide (PI) staining

Fluorescence-based viability analysis of RAW 264.7 cells was performed using a Calcein-AM/Propidium Iodide double staining kit (CA1630, Solarbio, UK), according to the manufacturer's instructions and a previously established protocol [29]. Live cells were stained with Calcein-AM, emitting green fluorescence, and dead cells were stained with PI, emitting red fluorescence.

### 2.6 Animal model of femoral biomaterial implantation

Eighteen male Sprague-Dawley rats (300 g, 8–12 weeks old) were anesthetized via intraperitoneal injection of Zoletil 5 (30 mg/kg) and xylazine hydrochloride (5 mg/kg). All the animal procedures were approved by the Institutional Animal Care and Use Committee and the local Ethics Committee (Approval number: GY2023-721) and adhered to institutional guidelines. Cylindrical material rods were implanted into the femoral medullary cavity of the rats. The surgical site was sutured and disinfected under sterile conditions. On postoperative day (D) 3, the rats were euthanized, and the bone marrow surrounding the implants was harvested for scRNA-seq.

### 2.7 scRNA-seq

The Gel Beads-in-Emulsion (GEM) Single Cell 3′ Reagent Kit was utilized to generate GEMs and introduce cell barcodes, followed by post-GEM reverse transcription cleanup and cDNA amplification. A 3ʹ gene-expression library was constructed prior to sequencing. Demultiplexing of cell barcodes was conducted using the MobiVision software pipeline (version 1.1, MobiDrop). Sequence reads were aligned to the reference genome and transcriptome using the STAR aligner (version 2.7.11b). To ensure comparability across samples, read downsampling was performed to normalize sequencing depth, generating a unified gene-cell count matrix for comprehensive transcriptional landscape analysis of the reparative tissue.

The R package Seurat was utilized for quality control, dimensionality reduction, and clustering of the scRNA-seq data [30]. Quality control filters retained cells with 200–6000 detected genes and mitochondrial gene content below 25 %. Data normalization was conducted via the SCTransform method to mitigate technical variation. Highly variable genes were identified using FindVariableFeatures(). To correct for batch effects arising from multiple samples or sequencing runs, we applied the Harmony algorithm integrated within Seurat for dataset integration and batch correction, improving clustering accuracy and biological signal recovery. Dimensionality reduction was performed through principal component analysis (PCA) followed by uniform manifold approximation and projection (UMAP) for visualization. Cell clustering was conducted using a shared nearest neighbor (SNN) modularity optimization-based algorithm, and clusters were annotated based on canonical marker genes. Differential gene expression analysis between clusters or conditions was carried out using the FindAllMarkers() function with the Wilcoxon rank-sum test, controlling for multiple comparisons. Cell-cell communication across distinct subpopulations was inferred using the CellChat package [31]. Finally, Kyoto Encyclopedia of Genes and Genomes (KEGG) and Gene Ontology (GO) enrichment analyses of differentially expressed genes (DEGs) were performed using the SangerBox online platform [32]. To ensure reproducibility, all analyses were conducted with fixed random seeds, and key findings were validated via subsampling and replication using independent datasets. Further analysis procedures have been added to the Supplementary Data.

### 2.8 Molecular docking and binding affinity analysis

Protein-protein docking for the CD99-CD99 homodimer and ICAM1-SPN heterocomplex was performed using ClusPro2.0 [33]. Initial structures were downloaded from the RCSB PDB database and prepared in PyMOL software through structural optimization and molecular dynamics simulations [34]. The docking employed Fast Fourier Transform (FFT)-based efficient global sampling of peptide recognition motifs, with binding poses ranked by weighted center scoring metrics. The dominant cluster conformation from each complex was selected for binding free energy prediction via MM/GBSA calculations.

### 2.9 Histological analysis

The femurs containing the implants were collected for decalcification, embedding, sectioning, and staining. Hematoxylin and Eosin (H&E) staining was performed to assess the cellular changes in the tissues surrounding the implants. For immunofluorescence analysis, a multiplex fluorescent immunohistochemistry mouse/rabbit kit (pH 9.0) (RS0037, ImmunoWay, USA) was used according to the manufacturer's instructions [35]. The following antibodies were sequentially applied: CD68 (1:5000, 66231-2-Ig, Proteintech, China), CD3E (1:300, bs-0765R, Bioss, China), CD99 (1:100, MA5-47656, Thermo Fisher Scientific, USA), ICAM1 (1:500, ab282575, Abcam, UK), SPN (1:100, A6412, ABclonal, USA), and CD34 (1:300, bs-0646R, Bioss, China), followed by DAPI for nuclear staining. The co-localization of these markers was assessed using ImageJ.

### 2.10 Real-time quantitative PCR

The Trizol kit extracts total RNA from peri-implant tissue. RNA was reverse transcribed to cDNA using the PrimeScriptTM RT kit (Takara Clontech, Kyoto, Japan). RT-qPCR was performed on the CFX Connect real-time system (BioRad, California, USA) using the SuperReal PreMix Plus (SYBR Green) kit (Takara Clontech, Kyoto, Japan). The thermal cycling conditions for PCR amplification were 9 °C for 5 min, 95 °C for 10 s, 60 °C for 30 s, 40 cycles, and then 40 °C for 20 min mRNA expression was normalized to GAPDH mRNA levels. Primers for real-time quantitative PCR (RT-qPCR) are listed in Supplementary Table S1.

### 2.11 Statistical analysis

All the values are presented as mean ± standard deviation. Statistical significance between the two groups was assessed using Student's *t*-test. Statistical analyses were conducted using GraphPad Prism 9.5 (GraphPad Software, CA, USA). Differences were considered statistically significant at *p* < 0.05.

## 3 Results

### 3.1 Characterization of the Ti and PEKK implants

**Fig. 1:**
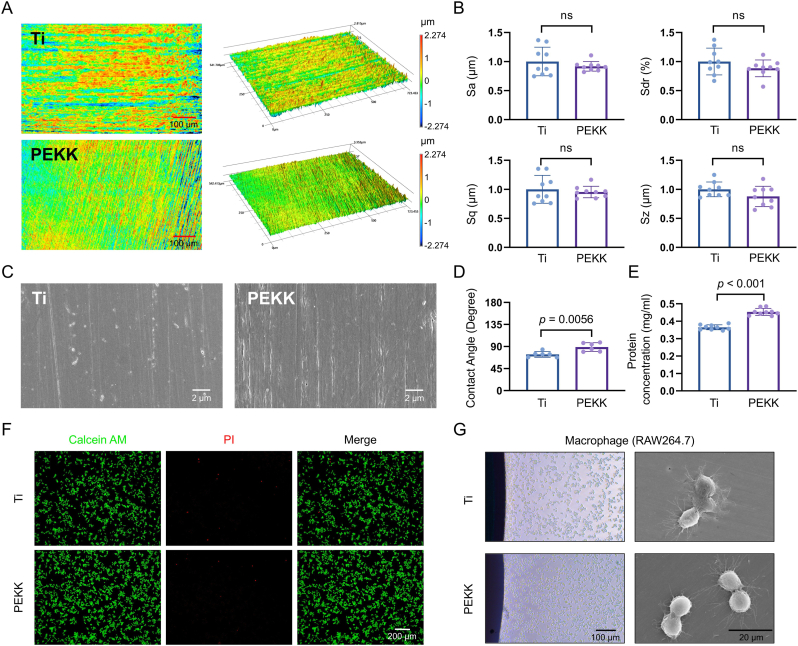
Characterization of the Ti and PEKK implants. (**A** and **B**) Surface roughness and corresponding quantitative analysis, including Sa (arithmetical mean height) and Sq (root mean square height). Scale bars: 100 μm. (**C**) Representative SEM images of the material surfaces. Scale bars: 2 μm. (**D**) Contact-angle measurements based on the droplet method, along with corresponding quantitative analysis. (**E**) Protein-adhesion amounts on the material surfaces. (**F**) Cytotoxicity assessment of specimen extracts based on AM/PI staining. Scale bars: 200 μm. (**G**) Direct cell contact and adhesion on material surfaces. Statistical significance was assessed using Student's *t*-test.

The surface roughness of the Ti and PEKK specimens was measured using a non-contact confocal laser scanning microscope at × 20 magnification. Parameters such as arithmetical mean height (Sa), root mean square height (Sq), developed interfacial area ratio (Sdr), and maximum height (Sz) were calculated. The results revealed comparable surface morphologies between the two types of specimens, with no statistically significant differences (Fig. 1A and B). The representative SEM imaging further confirmed that the surface morphologies of the two specimens were similar, showing no statistically significant topographical differences (Fig. 1C). Contact angle measurements of these two materials showed that Ti had an average contact angle of 73.9° ± 1.6°, whereas PEKK displayed an average contact angle of 90.6° ± 1.2° (sessile drop method, n = 6), suggesting favorable wetting characteristics of Ti (Fig. 1D). Elemental composition of Ti and PEKK surfaces was analyzed using EDS mapping. The Ti surface exhibited a large amount of uniformly distributed Ti along with small amounts of alloying elements (Fig. S1A–C). The PEKK surface contained a large amount of uniformly distributed C, along with small amounts of O, F, S, and Cl (Fig. S1A, D and E).

Additionally, protein adsorption analysis revealed that the protein concentration on Ti surfaces measured 0.36 ± 0.01 mg/mL, while PEKK surfaces adsorbed 0.45 ± 0.02 mg/mL of protein, indicating superior protein-binding capacity of PEKK's surface characteristics (*p* < 0.001) (Fig. 1E). Macrophages were cultured in extracts of Ti or PEKK specimens. Both groups showed minimal cell death, indicating low cytotoxicity for both materials (Fig. 1F). Cell-adhesion experiments revealed abundant cells both on and around the surfaces of the Ti and PEKK specimens, indicating that cells can effectively adhere to these materials (Fig. 1G).

### 3.2 Characterization of the bone-marrow microenvironment post-implantation

**Fig. 2:**
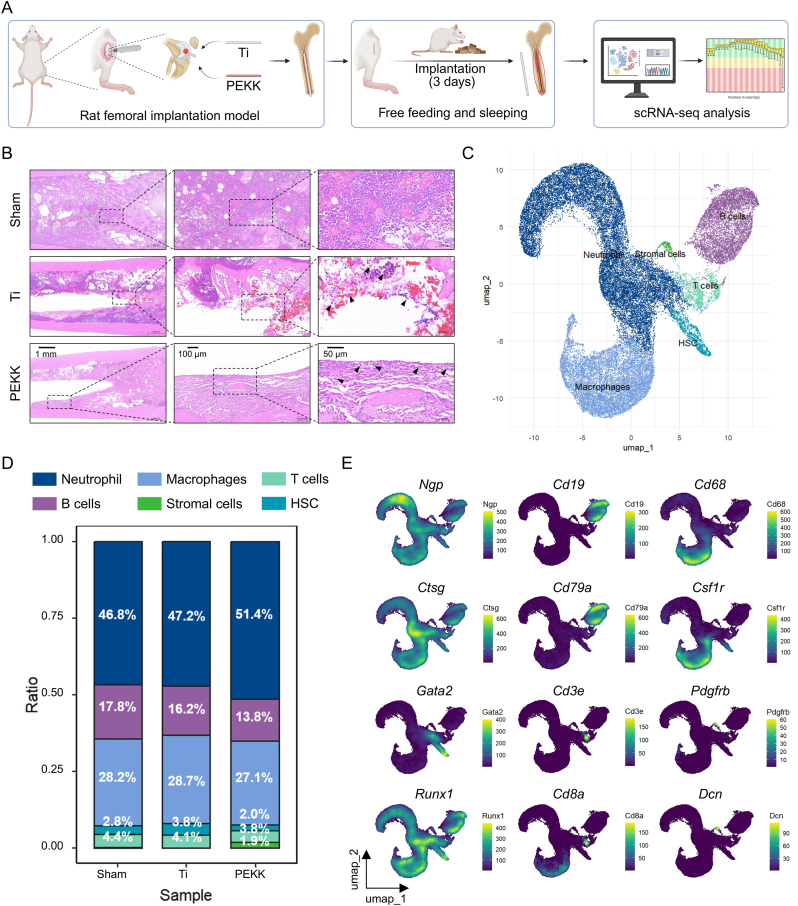
Construction of the early bone-marrow cell atlas following femoral Ti or PEKK implantation. (**A**) Establishment of the animal model of femoral biomaterial implantation and the experimental workflow. (**B**) H&E staining of the bone marrow surrounding each implant. The structure indicated by the arrow is an immune cell. Scale bars: 1 mm, 100 μm, and 50 μm. (**C**) Bone-marrow cell atlas post-implantation. (**D**) Proportional distributions of cell types and (**E**) Expression patterns of classical cell marker genes in the implant groups.

To investigate early responses in the bone-marrow microenvironment following Ti and PEKK implantation, a rat femoral implantation model was established. The bone-marrow surrounding the implants was collected on day 3 post-implantation for scRNA-seq and histological analysis (Fig. 2A and S2A). H&E staining of bone marrow revealed scattered macrophage-like cells around both the Ti and PEKK implants (Fig. 2B). H&E staining revealed scattered macrophages surrounding the implants, with no significant differences in overall cellular composition between the two implants. Quality control was performed on the scRNA-seq data to remove multiplets and low-quality cells, retaining a total of 42,299 cells from the bone marrow tissue surrounding the implants (Fig. S2B–E). Principal component analysis was used for dimensionality reduction, and batch effects were corrected using Harmony. A total of 21 distinct cell clusters were identified, and a cell atlas was constructed based on the DEGs and following marker genes: neutrophils (*Ngp* and *Ctsg*), B cells (*CD19* and *CD79a*), macrophages (*CD68* and *Csf1r*), HSCs (*Gata2* and *Runx1*), T cells (*Cd3e* and *Cd8a*), and stromal cells (*Pdgfrb* and *Dcn*) (Fig. 2C–E and S2F-H). Subsequently, the cell proportions in the sham, Ti, and PEKK groups were visualized to assess the differences in cell composition across the different implant conditions. Although the PEKK group was slightly different from the sham and Ti groups in cell composition, the differences were not statistically significant (Fig. 2D). Thus, both the Ti and PEKK implants preserved the overall cellular architecture of femoral bone marrow, as the number of distinct clusters and the distribution of major cell types remained comparable to those of the sham.

### 3.3 CD99-CD99 crosstalk between macrophages and T cells evokes an anti-inflammatory response in bone marrow following Ti implantation

**Fig. 3:**
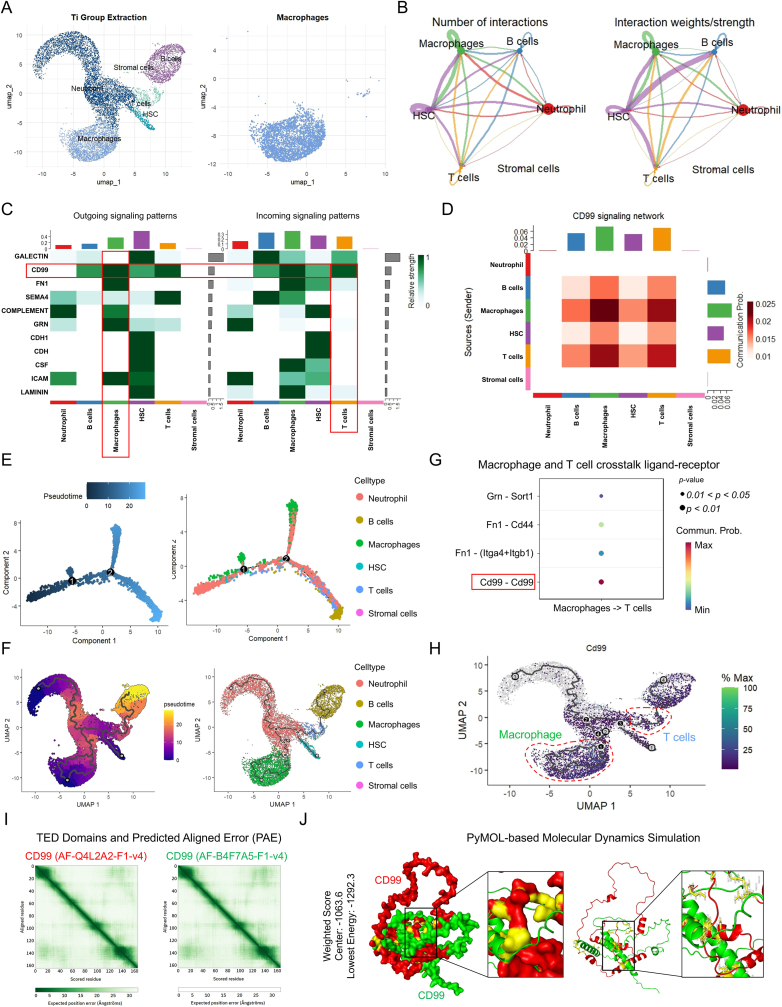
CD99-CD99 crosstalk between macrophages and T cells around the Ti-based implant. (**A**) Isolation of the macrophages surrounding the implant. (**B**) Number of interactions and interaction strengths, and (**C**) outgoing and incoming signaling patterns among different cell types. (**D**) Heatmap showing the interaction strength within the CD99 signaling network. (**E** and **F**) Cellular-trajectory analysis and atlas mapping, based on Monocle2 and Monocle3, respectively. (**G**) Candidate ligand-receptor pairs. (**H**) Expression patterns of the candidate ligand-receptor pairs across cell populations. (**I** and **J**) Molecular dynamics simulations and binding energy prediction.

To investigate macrophage interactions within the bone-marrow microenvironment, macrophages adjacent to the Ti implant were isolated for further analysis (Fig. 3A). Cell-cell interaction analysis indicated that these macrophages engaged in crosstalk with HSCs, T cells, B cells, and neutrophils (Fig. 3B and S3A, B). Evaluation of interaction strength revealed that macrophages displayed strong outgoing signaling through CD99, whereas T cells exhibited the strongest incoming signaling patterns among the cell types analyzed (Fig. 3C and S3C). Within the CD99 signaling network, macrophages and T cells displayed robust interactions (Fig. 3D and S3D, E).

To characterize the temporal relationship between macrophages and T cells, Monocle2 was utilized to construct a cell-differentiation trajectory, which showed that macrophages emerged first, followed by T cells (Fig. 3E). Mapping this differentiation trajectory onto the cellular atlas by using Monocle3 confirmed the emergence of macrophages before T cells, suggesting a potential regulatory role of macrophages in T-cell differentiation (Fig. 3F). To elucidate how macrophages regulate T cells, ligand-receptor pairs between macrophages and T cells were evaluated, revealing *Cd99*-*Cd99* as the strongest interacting pair (Fig. 3G). Subsequent mapping of *Cd99* expression onto the cell atlas showed that *Cd99* was predominantly expressed in both macrophages and T cells, suggesting that CD99-CD99 interactions mediate the crosstalk between the two cell types (Fig. 3H).

Molecular dynamics simulations and binding energy analysis suggested potential protein-protein interactions between CD99 homodimers. The data indicated a possible molecular binding interface (yellow region), with a weighted center score of −1063.6 and lowest energy state of −1292.3 (Fig. 3I and J). These negative energy values may imply favorable intermolecular forces between CD99 monomers, pointing to potential dimerization behavior.

**Fig. 4:**
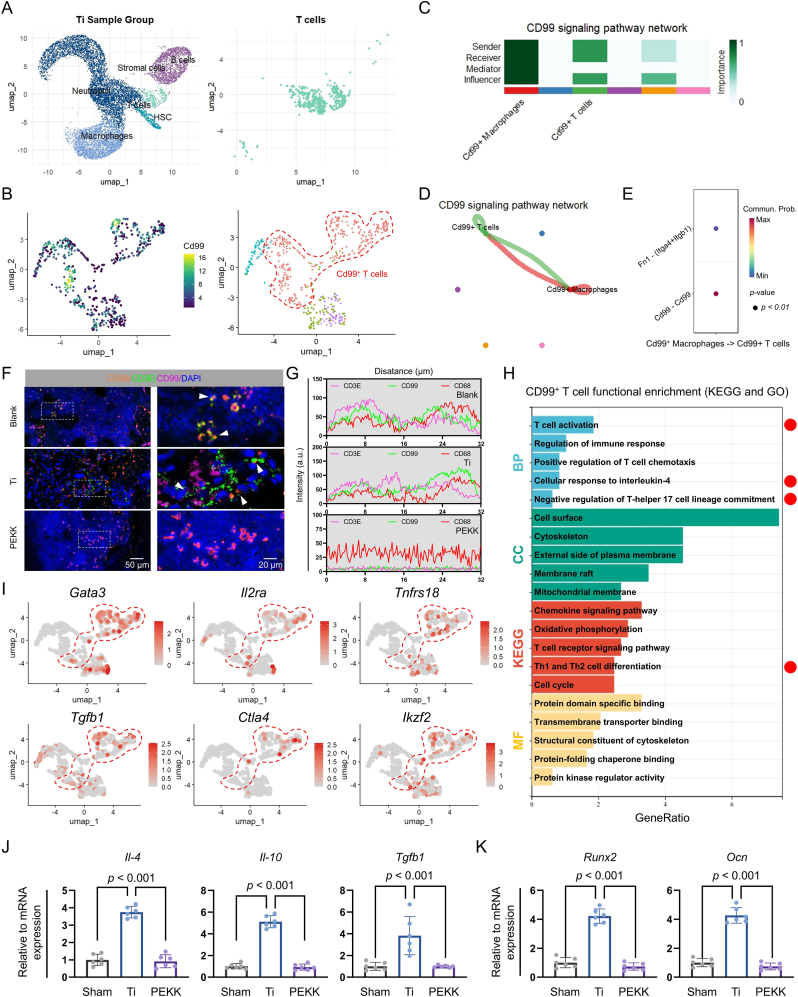
The CD99-CD99 crosstalk between macrophages and T cells around the Ti implant evokes an anti-inflammatory response. (**A**) Isolation of the T cells surrounding the Ti implant. (**B**) Identification and isolation of the *Cd99*^+^ T cell subpopulation. (**C** and **D**) Interaction network between *Cd99*^+^ macrophages and *Cd99*^+^ T cells. (**E**) Interaction strengths of the candidate ligand-receptor pairs between macrophages and T cells. (**F** and **G**) Multiplex co-localization analyses of the candidate ligand-receptor pairs. Scale bars: 50 μm and 20 μm. (**H**) KEGG and GO (BP, CC, and MF) enrichment analysis of the differentially expressed genes in the *Cd99*^+^ T cells surrounding the Ti implant. (**I**) Cellular localization of anti-inflammatory genes. (**J** and **K**) Assessment of transcriptional levels for anti-inflammatory-associated and osteogenic genes by Real-time quantitative PCR (RT-qPCR). Statistical analysis was performed with one-way ANOVA.

Next, T cells surrounding the Ti implant were isolated, and the *Cd99*^+^ sub-population was identified via CellChat (Fig. 4A and B, and S4A-C). Signaling analysis of *Cd99*^+^ macrophages and *Cd99*^+^ T cells revealed that the interaction between these two cell types was the strongest, with *Cd99*-*Cd99* identified as the predominant ligand-receptor pair, consistent with previous findings (Fig. 4C–E and S4D). To validate the CD99-CD99 crosstalk between macrophages and T cells, their localization patterns around the Ti implant were evaluated. To this end, CD68 and CD3E were used as markers of macrophages and T cells, respectively. Immunofluorescence analysis showed clear colocalization of CD68, CD3E, and CD99 (Fig. 4F and G). These findings support the CD99-CD99 crosstalk between macrophages and T cells around the Ti implant.

To explore the role of *Cd99*^+^ T cells in the post-implant bone-marrow microenvironment, DEGs were identified using the FindMarkers function in Seurat, followed by KEGG and GO (Biological Process, BP; Cellular Component, CC; and Molecular Function, MF) enrichment analyses (Fig. S4E and F). The significantly enriched pathways included cellular response to interleukin-4, negative regulation of T-helper 17 cell lineage commitment, T cell activation, and Th1 and Th2 cell differentiation (Fig. 4H). Additionally, anti-inflammatory genes were highly expressed in *Cd99*^+^ T cells, further suggesting the anti-inflammatory role of these cells (Fig. 4I). RT-qPCR analysis of peri-implant tissues revealed significant transcriptional upregulation of anti-inflammatory genes *Il-4*, *Il-10*, and *Tgfb1* in the Ti group compared to both sham group and PEKK group (Fig. 4J). Similarly, early-phase osteogenic genes *Runx2* and *Ocn* exhibited markedly elevated expression levels surrounding Ti implants (Fig. 4K). These findings suggest that CD99^+^ T cells may contribute to the titanium-associated anti-inflammatory response and subsequent osteogenesis.

### 3.4 ICAM1-SPN crosstalk between macrophages and HSCs causes HSCs necroptosis in bone marrow following PEKK implantation

**Fig. 5:**
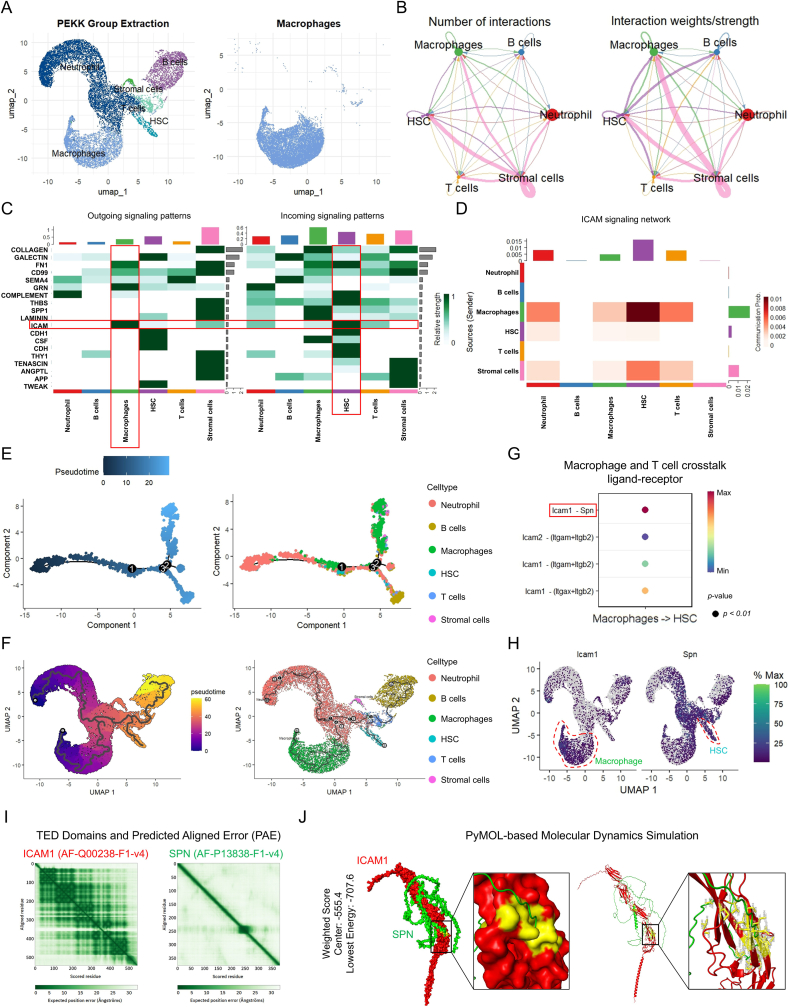
ICAM1-SPN crosstalk between macrophages and HSCs around the PEKK implant. (**A**) Isolation of the macrophages surrounding the implant. (**B**) Number of interactions and interaction strengths, and (**C**) Outgoing and incoming signaling patterns among different cell types. (**D**) Heatmap showing the interaction strength within the ICAM signaling network. (**E** and **F**) Cellular-trajectory analysis and atlas mapping, based on Monocle2 and Monocle3, respectively. (**G**) Candidate ligand-receptor pairs. (**H**) Spatial expression patterns of the candidate ligand-receptor pairs across cell populations. (**I** and **J**) Molecular dynamics simulations and binding energy prediction.

To investigate the crosstalk between macrophages and other cell types in the bone-marrow microenvironment following PEKK implantation, macrophages were isolated and analyzed from the data set (Fig. 5A). Cell-cell interaction analysis and outgoing–incoming signaling patterns revealed that macrophages communicate with HSCs via the ICAM signaling (Fig. 5B, C, and S5A-C). The ICAM signaling network further demonstrated a strong interaction between macrophages and HSCs (Fig. 5D and S5D, E). To characterize their temporal relationship, a Monocle2-based trajectory analysis was conducted. The results showed that macrophages emerged before HSCs, whose numbers subsequently declined (Fig. 5E). Consistently, Monocle3-based trajectory mapping on the cell atlas confirmed this sequential emergence, with macrophages preceding HSCs (Fig. 5F). These findings suggest that macrophages modulate HSCs’ function in PEKK-implanted bone marrow.

To further explore how macrophages regulate HSCs, a ligand-receptor interaction analysis between these two cell types was performed, identifying *Icam1*-*Spn* as the strongest interacting pair (Fig. 5G). Trajectory mapping revealed that *Icam1* was primarily expressed in macrophages, whereas *Spn* was predominantly expressed in HSCs, suggesting that their interaction occurs via ICAM1-SPN coupling (Fig. 5H). Molecular dynamics simulations and binding energy prediction were employed to characterize the ICAM1-SPN ligand-receptor pair. The results demonstrated interlocking structural complementarity between ICAM1 and SPN proteins, with a distinct potential binding interface (yellow region). The weighted center score (−555.4) and lowest binding energy (−707.6) suggested thermodynamically favorable binding (Fig. 5I and J). These computational insights imply that macrophage-mediated signaling via the ICAM1-SPN axis may regulate HSCs functionality.

**Fig. 6:**
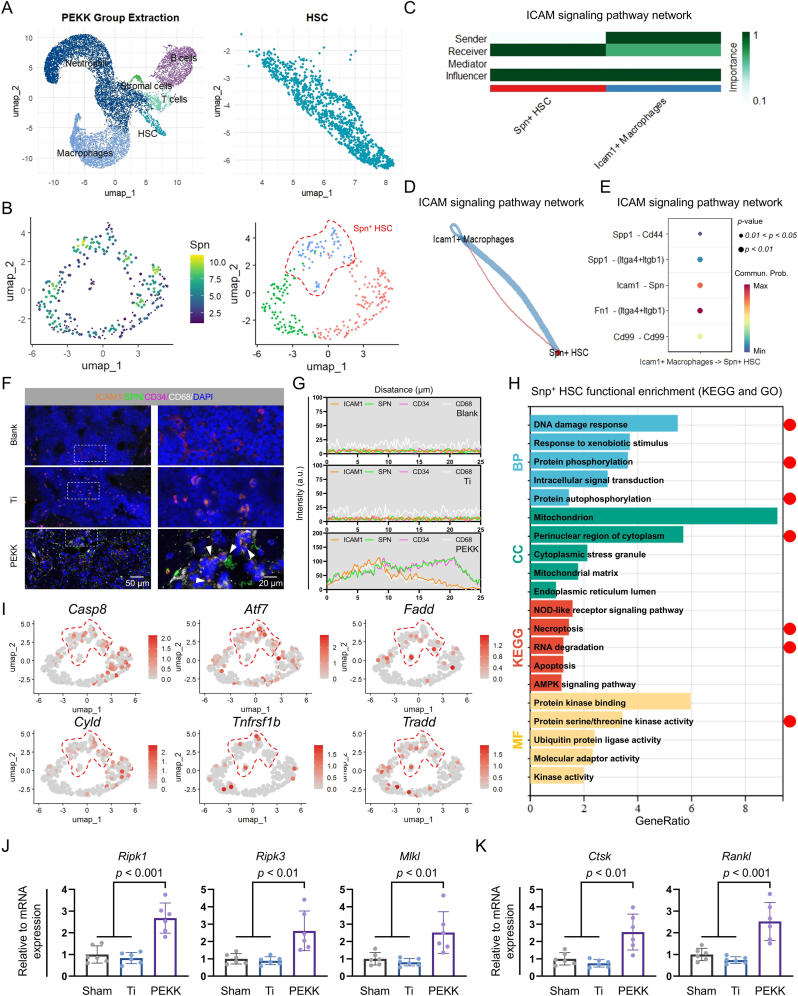
The ICAM1-SPN crosstalk between macrophages and HSCs around the PEKK implant induces HSCs necroptosis. (**A**) Isolation of the HSCs surrounding the implant. (**B**) Identification and isolation of the *Spn*^+^ HSCs subpopulation. (**C** and **D**) Interaction network between Icam1+ macrophages and *Spn*^+^ HSCs. (**E**) Interaction strength of candidate ligand-receptor pairs between macrophages and HSCs. (**F** and **G**) Colocalization analysis of ligand-receptor pairs. Scale bars: 50 μm and 20 μm. (**H**) KEGG and GO (BP, CC, and MF) enrichment analyses of the DEGs in the *Spn*^+^ HSCs surrounding the implant. (**I**) Spatial expression patterns of necroptosis-related genes in HSCs. (**J** and **K**) Assessment of transcriptional levels for necroptosis-associated and osteoclast-related genes by quantitative real-time PCR (RT-qPCR). Statistical analysis was performed with one-way ANOVA.

Building on these findings, HSCs surrounding PEKK implants were isolated, and the *Spn*^+^ sub-population was identified (Fig. 6A and B, and S6A-C). Signaling analysis between *Icam1*^+^ macrophages and *Spn*^+^ HSCs showed strong intercellular communication, with *Icam1*-*Spn* exhibiting high interaction intensity, further supporting the proposed mechanism (Fig. 6C–E and S6D). To validate this crosstalk, the macrophages and HSCs around the PEKK implant were evaluated for their expression of ICAM1 and SPN. Immunofluorescence analysis revealed partial co-localization between ICAM1 and the macrophage marker CD68, while SPN was expressed on the surface of HSCs (CD34^+^) with overlapping signal distribution (Fig. 6F). Fluorescence co-localization analysis further demonstrated co-expression of ICAM1 with CD68 and SPN with CD34 in the PEKK group, along with spatial overlap between ICAM1 and SPN (Fig. 6G). This collective evidence suggests potential ligand-receptor interactions within the ICAM1-SPN pair.

To explore the functional regulation of HSCs by macrophages following PEKK implantation, DEGs in *Spn*^+^ HSCs were identified and analyzed via KEGG and GO (BP, CC, and MF) enrichment analyses (Fig. S6E and F). The results revealed significant enrichment in pathways associated with necroptosis, DNA damage response, protein phosphorylation, perinuclear cytoplasmic localization, and serine/threonine kinase activity (Fig. 6H). To validate these findings, the expression of necroptosis-related genes (*Casp8*, *Atf7*, *Fadd*, *Cyld*, *Tnfrsf1b*, and *Tradd*) was examined and found to be upregulated (Fig. 6I). These results suggest that HSCs undergo necroptosis-related alterations following crosstalk with macrophages in PEKK-implanted bone marrow.

RT-qPCR analysis of peri-implant tissues revealed significant transcriptional upregulation of necroptosis-associated genes (*Ripk1*, *Ripk3*, *Mlkl*) in the PEKK group compared to sham and Ti controls (Fig. 6J). Concurrently, elevated expression of osteoclast-related genes *Ctsk* and *Rankl* (*Tnfsf11*) was observed (Fig. 6K). These findings suggest that early-phase macrophage-HSCs interactions within the PEKK-implanted bone marrow microenvironment may trigger necroptotic pathways and subsequently alter osteoclastic activity.

## 4 Discussion

The early biological response to implanted biomaterials is a critical determinant of both biocompatibility and clinical performance. As a highly dynamic cellular ecosystem, the bone-marrow microenvironment plays a critical role in maintaining hematopoietic homeostasis and orchestrating immune regulation. In this study, scRNA-seq and validation analyses were used to systematically elucidate the early molecular and cellular remodeling of the bone-marrow microenvironment following Ti and PEKK implantation, offering novel insights into the mechanisms underlying biomaterial–host interactions.

Comparative analysis revealed that both the Ti and PEKK implants have excellent biocompatibility profiles, with cell viability and subset distributions meeting ISO 10993 criteria for orthopedic biomaterials. The intrinsic physicochemical stability of PEKK-reflected by the absence of detectable degradation products throughout the study-contributes to its favorable biocompatibility [6,36]. Interestingly, Ti exhibits comparable performance, primarily through distinct immunomodulatory mechanisms.

Although H&E staining revealed similar macrophage recruitment patterns around both implants in the early stages, scRNA-seq revealed material-specific immune modulation profiles. The Ti group exhibited notable reprogramming of *Cd99*^+^ T cell subsets, with transcriptional profiles consistent with Th2 polarization—phenotypically linked to the observed *in vivo* anti-inflammatory responses of Ti implants [37,38]. This finding aligns with recent studies indicating that roughened hydrophilic Ti surfaces promote M2-like macrophage polarization, characterized by increased IL-4 and IL-13 secretion, which suppresses pro-inflammatory responses and promotes tissue repair [39]. The elevated expression of immune-regulatory genes (e.g., IL-10, TGF-β) around Ti implants may induce B-cell immunomodulatory functions in bone repair [40]. In contrast, pronounced neutrophil infiltration in the PEKK group might exacerbate local inflammation.

These cytokines not only suppress inflammatory responses but also promote bone integration by creating a pro-repair microenvironment. The phenomenon of Th2 cell polarization aligns with the classical theory-Th2 cells promote tissue regeneration through related interleukins, with upregulation of gene expression (e.g., *Gata3* and *Tgfb1*). Together, these processes establish an immunosuppressive environment favorable for osseointegration [41,42]. The identification of specific T cell subsets holds significant biological relevance: the upregulation of IL-10 and TGFB1 on their surfaces inhibits excessive immune activation and promotes tissue proliferation via the cytokine network [43]. The expression profiles of functional genes (anti-inflammatory, osteogenic, and necroptosis) further indicate functional changes in the surrounding tissue during the early stages of Ti and PEKK implantation. This immune cell subset may form a comprehensive axis involving Ti-based material surface characteristics, cellular interaction, and tissue repair, integrating innate immune signals with adaptive immune responses.

This study further explored the intricate mechanisms governing HSCs regulation within PEKK microenvironments, uncovering several key findings that merit further investigation. The predominant influence of PEKK on hematopoietic homeostasis, in contrast to the modulation of adaptive immunity by Ti, underscores a distinct paradigm in material-host interactions [44,45]. Central to this observation is the macrophage-HSCs communication axis, mediated by ICAM1-SPN signaling, which emerged as a critical determinant of HSCs fate in PEKK-implanted environments. Hematopoietic homeostasis, defined as the dynamic balance of blood cell production and function, is essential for immune competence and tissue repair capacity [46]. In the context of osseointegration, the immune response-particularly macrophage-mediated tissue remodeling-is closely linked to hematopoietic homeostasis. Disruptions in HSCs function or niche integrity can impair macrophage differentiation and activation, potentially compromising osseointegration outcomes [47]. Furthermore, the surface characteristics of PEKK-based implants, through their modulation of immune cell behavior and cytokine secretion, may indirectly influence hematopoietic homeostasis and bone regeneration [48,49]. For PEKK implants, the early immune response involving *Icam1*^+^ macrophages inducing HSCs necroptosis can be effectively modulated. A sustained-release coating loaded with IL-10 or ICAM1 inhibitors specifically targets SPN^+^ macrophages, suppressing their activity while regulating early immune crosstalk [50,51]. This optimization strategy demonstrates significant potential to enhance bone repair efficiency and facilitate clinical translation.

Our results showed that macrophages, functioning as early responders to biomaterial implantation, exhibited increased interactions with HSCs in PEKK-implanted bone marrow compared to Ti-based implants. This intercellular communication was not a passive occurrence but instead involved active signaling through the ICAM1-SPN ligand-receptor pair, which has previously been associated with immune-cell adhesion and migration [52,53]. The upregulation of necroptosis-related genes and activation of DNA-damage response pathways in HSCs adjacent to macrophages indicates a dynamic interaction in which macrophages may induce specific stress responses in HSCs [54,55]. This finding aligns with emerging concepts that macrophages serve as both sensors and effectors of tissue homeostasis, influencing stem-cell behavior in response to microenvironmental signals [56,57].

The limited osteointegration capacity of PEKK, despite its demonstrated biocompatibility, may be attributed to its insufficient regulation of chronic inflammation. The occurrence of this chronic inflammation may be associated with sustained high expression of IL-1β or activation of the NLRP3 inflammasome [58]. Although macrophage-driven immune adaptations are essential for initiating tissue responses, dysregulation of these mechanisms can destabilize the hematopoietic niche and hinder bone repair [59,60]. The failure to resolve the inflammation around PEKK implants may trigger a feedback loop in which sustained macrophage activation and HSCs stress compromise regenerative outcomes. This hypothesis is supported by the distinct transcriptional profile observed in HSCs near the PEKK implant, marked by the activation of stress-response pathways that may impair cellular functionality. Such effects may be linked to the intrinsic bioinertness of PEKK, characterized by its hydrophobic surface properties (water contact angle >90°), which impairs osteogenesis through two synergistic mechanisms: (1) inadequate establishment of an immunoregulatory network required for inflammation resolution, and (2) sustained inflammatory signaling that not only maintains M1 macrophage polarization but also promotes osteoclastogenesis via RANKL upregulation [[61], [62], [63]]. Recent studies demonstrate that plasma treatment combined with sulfonation to create porous structures on PEKK surfaces, coated with magnesium chondroitin sulfate (MgCS), reduces IL-1β and TNF-α release, mitigating acute inflammation [64]. Hao Gu's team employed femtosecond laser etching-sulfonation to construct biomimetic micro-nano structures, simulating the MET signaling pathway, reprogramming macrophage metabolism, and significantly improving osseointegration efficiency in osteoporotic models [65]. Integrated with our findings on early immune crosstalk targets in bone implants, material modifications can optimize the bone immune microenvironment, providing potential targets for osteogenic differentiation [66,67].

This study provides valuable insights into the early molecular and cellular remodeling following Ti and PEKK implantation; however, several limitations should be acknowledged. Specifically, the use of animal models and restricted early observation windows limit the assessment of human immune responses and long-term outcomes; the lack of specific subcellular inhibitors and the low efficiency of plasmid delivery in bone tissue hinder the consistent acquisition of stable animal functional performance; and single-cell transcriptomics lacks spatial resolution, preventing the delineation of cell-cell interactions at the implantation interface. Based on these limitations, future research will integrate spatial multi-omics and gene-edited animal analyses to map the fine-grained spatial intercellular communication atlas within the local microenvironment. This will inform the design of surface-modified implants incorporating immunomodulatory strategies (e.g., CD99-mimetic peptides or ICAM1-blocking coatings). Subsequent long-term preclinical validation in large animal models will evaluate immune responses (via multi-omics monitoring) and osseointegration (via μCT and biomechanical testing). This integrated approach aims to facilitate the design and clinical translation of next-generation biomaterials with optimized osteoimmunomodulatory properties.

## 5 Conclusion

This study examined the early immune responses to Ti and PEKK implants within the bone marrow microenvironment. Ti-based implants facilitated the establishment of an anti-inflammatory microenvironment and promoted osseointegration by inducing M2-like macrophage polarization and Th2 cell polarization, reflecting high biocompatibility and strong clinical potential. In comparison, PEKK implants, although biocompatible, demonstrated bioinert characteristics and failed to resolve chronic inflammation effectively. These deficiencies resulted in persistent macrophage activation and HSCs stress, probably disrupting hematopoietic homeostasis and delaying bone repair. These findings offer novel insights into biomaterial design and underscore the critical role of immunomodulatory properties in enhancing the functional performance of implanted biomaterials.

## CRediT authorship contribution statement

**Jiannan Zhou:** Writing – review & editing, Writing – original draft, Formal analysis, Data curation, Conceptualization. **Huibin Liang:** Writing – original draft, Methodology, Investigation, Data curation. **Jiahao Chen:** Writing – review & editing, Methodology. **An Li:** Writing – review & editing, Formal analysis. **Jingtao Dai:** Writing – review & editing, Methodology. **Ping Li:** Writing – review & editing, Writing – original draft, Supervision, Methodology, Funding acquisition, Formal analysis, Conceptualization.

## Declaration of competing interest

The authors declare that they have no known competing financial interests or personal relationships that could have appeared to influence the work reported in this paper.

## Data Availability

Data will be made available on request.
