# Supplementary material for: Macrophage-related immune responses to polyetherketoneketone bone implants: Single-cell transcriptome analysis
Source: Mater Today Bio. 2025 Aug 28;35:102257. doi: 10.1016/j.mtbio.2025.102257 (PMC12859614; doi:10.1016/j.mtbio.2025.102257)
Supplement: Multimedia component 1 [file mmc1.docx]

**Macrophage-Related Immune Responses to Polyetherketoneketone Bone Implants: Single-Cell Transcriptome Analysis**

**1. Supplementary Materials and Methods**

1.1 Single-cell communication analysis

Intercellular communication networks were analyzed using the CellChat pipeline. Initial processing of raw single-cell transcriptomic data included normalization via the SCTransform method and batch correction using the Harmony algorithm, implemented through the Seurat package. Following hierarchical clustering to identify cellular subpopulations, cell types were annotated by referencing the rat bone marrow atlas dataset. For cell-cell communication analysis, the CellChat platform was employed, leveraging its curated CellChatDB database to quantify interaction strengths between cell clusters through a communication probability model [1, 2]. This model was optimized via maximum likelihood estimation, with interaction scores derived from the geometric mean of ligand and receptor expression levels. To control false positives, 1,000 permutation tests were performed to filter significant pathways (FDR-adjusted *p* < 0.05), and hub cell populations were identified through network topology analysis (betweenness centrality, closeness centrality).

1.2 Functional enrichment analysis

The Database for Annotation, Visualization, and Integrated Discovery (DAVID; *http://david.abcc.ncifcrf.gov/*) was employed to perform Gene Ontology (GO) and Kyoto Encyclopedia of Genes and Genomes (KEGG) enrichment analyses, enabling functional classification and biological interpretation of differentially expressed genes (DEGs) [3-5]. A statistical significance threshold of *p* < 0.05 was applied to identify significantly enriched terms.

1.3 Monocle2-based pseudotime analysis

Pseudotemporal ordering of single cells was performed using the Monocle2 package [6]. Raw UMI counts were normalized via log_2_ (CPM+1) transformation with pseudocount adjustment. Highly variable genes were selected based on dispersion analysis, retaining genes with dispersion values exceeding 1.5-fold the mean dispersion across all detected genes. Dimensionality reduction was achieved using the DDRTree algorithm, which constructs a reduced-dimensional space (q = 10) through reversed graph embedding, followed by projection into a two-dimensional manifold for trajectory visualization. Cell ordering along the pseudotime axis was optimized by minimizing spanning tree complexity, with branch detection sensitivity parameterized at 1e-5. Branch-dependent differentially expressed genes were identified via the BEAM (Branch Expression Analysis Modeling) test, employing a negative binomial generalized additive model with significance thresholds set at q-value < 0.01 and fold-change > 1.5. Parameter stability was validated through 10 cycles of 5-fold cross-validation on randomly subsampled cells (5% of the total population), ensuring trajectory robustness against stochastic sampling effects during pseudotemporal trajectory construction.

1.4 Monocle3-based trajectory analysis

Parallel trajectory inference was conducted using Monocle3 with enhanced topological feature detection [6, 7]. The input matrix underwent library-size normalization followed by PCA decomposition retaining the top 30 principal components. Cellular manifolds were constructed through UMAP embedding (n.neighbors = 50, min.dist = 0.1) optimized for transcriptional topology preservation. Leiden graph-based clustering at a resolution of 0.01 identified discrete cell states, from which principal graphs were derived using a modular formulation of reversed graph embedding. Pseudotime values were computed by calculating geodesic distances from user-defined root nodes, with branch points automatically detected through curvature analysis of the embedded graph. Spatial autocorrelation patterns along trajectories were quantified using Moran’s I statistic, where genes demonstrating I > 0.25 and false discovery rate (FDR) < 5% were designated as significantly branch-associated. Computational reproducibility was assessed through 100 bootstrap iterations, with 95% confidence intervals confirming trajectory stability across subsampled datasets (10% variation tolerance in pseudotime ordering). UMAP trajectory curves were visualized to represent pseudotemporal progression.

**2. Supplementary Figure**


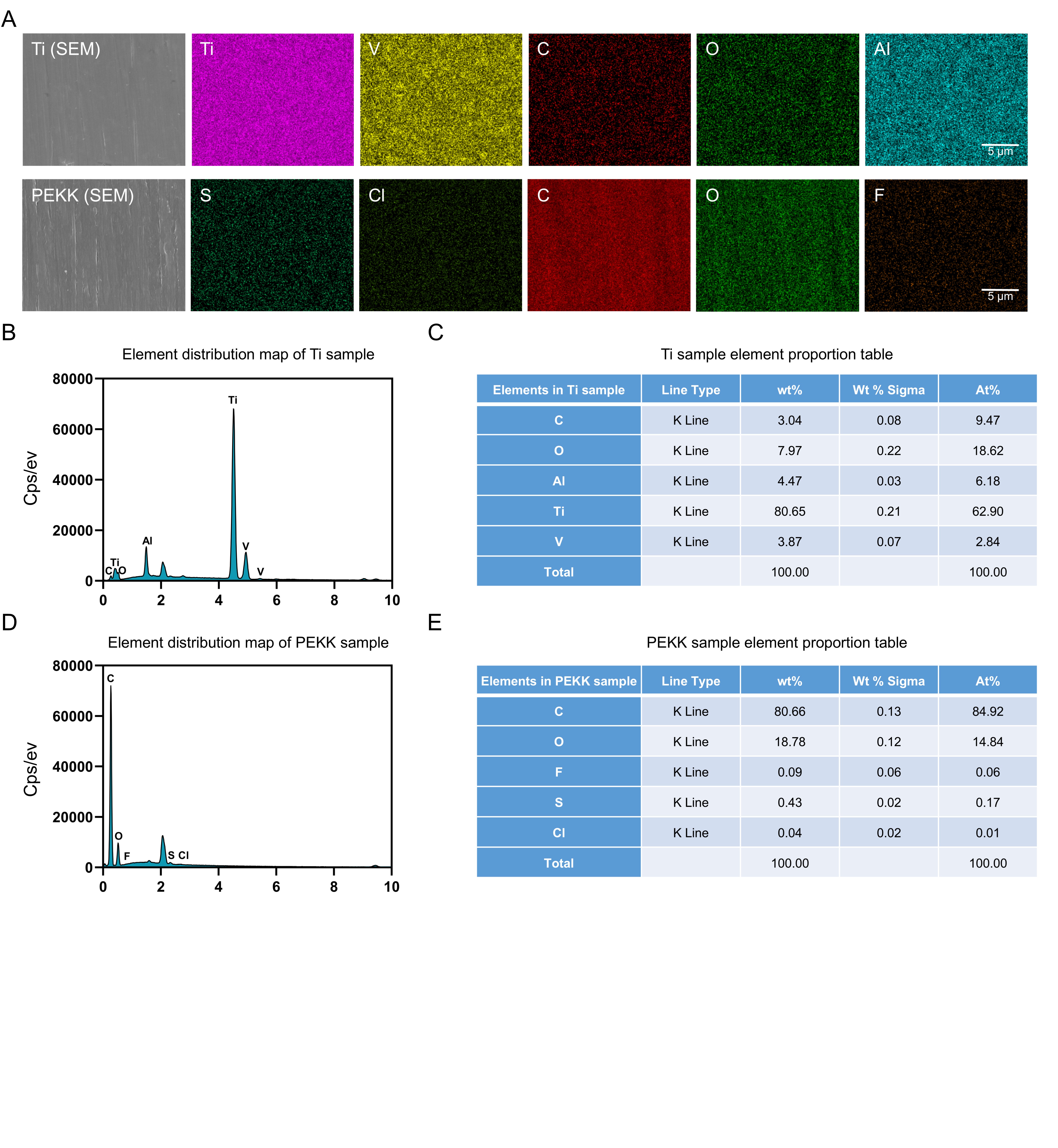


**Fig. S1** Characterization of Ti and PEKK materials. (**A**) Energy-dispersive X-ray spectroscopy (EDS) elemental mapping of designated regions on the material surfaces. Scale bars: 5 μm. (**B** and **C**) Quantitative analysis of surface roughness parameters: Sdr (Developed interfacial area ratio); Sz (Maximum height). EDS analysis of elemental composition in designated regions on the Ti surface. (**D** and **E**) EDS analysis of elemental composition in designated regions on the PEKK surface.


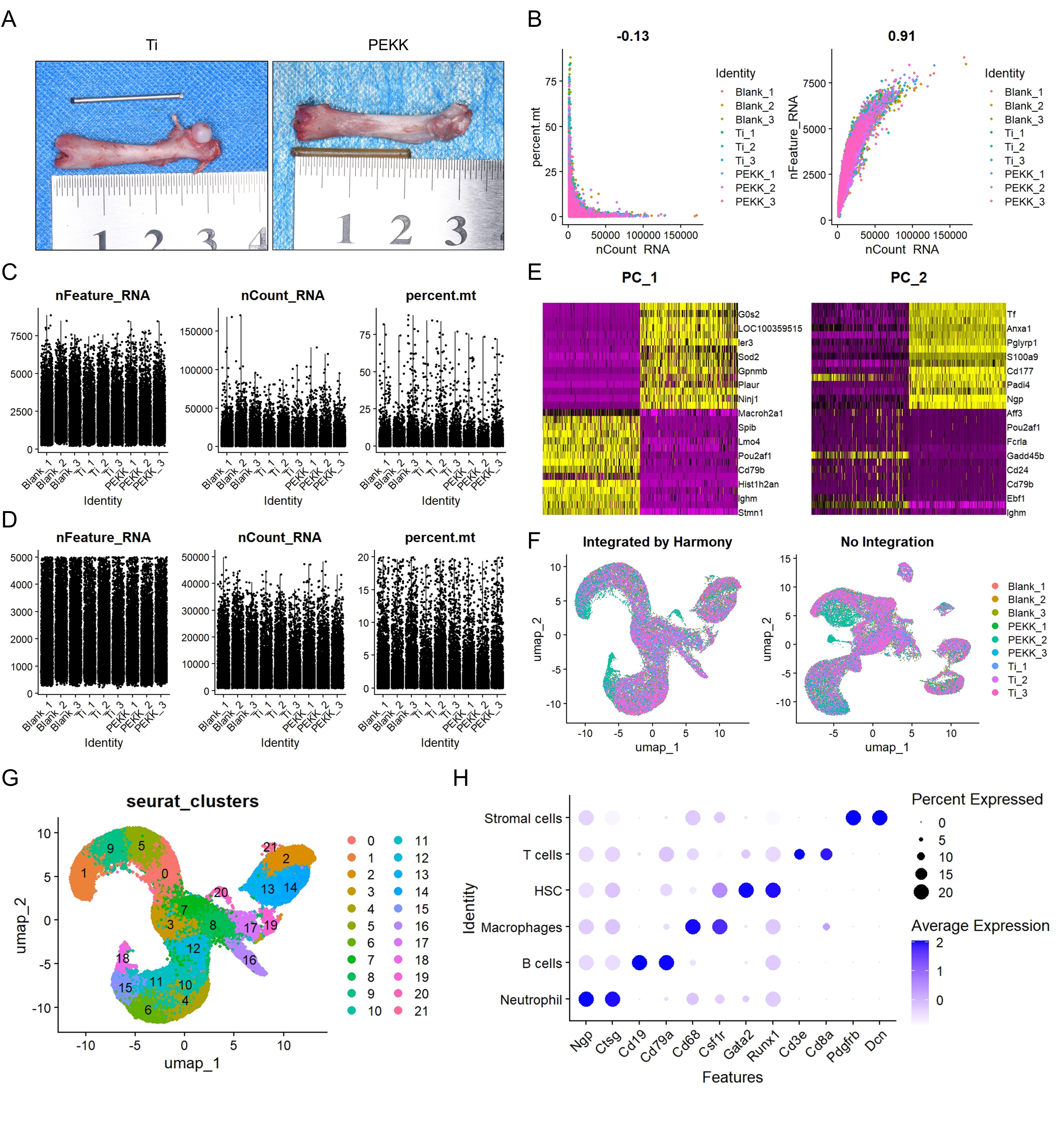


### Fig. S2 Early-stage implantation model construction and scRNA-seq dataset integration analysis of Ti and PEKK. (A) Images of rat femurs and extramedullary implants after implantation. (B-D) Gene-sample association analysis and preprocessing of the scRNA-seq dataset. (E) Clustering of differentially expressed genes after dimensionality reduction. (F) Integration and batch effect correction of the scRNA-seq dataset using Harmony, along with the corresponding cell distribution before and after correction. (G) Clustering analysis of cell populations. (H) Identification of cell types using classical marker genes.

**
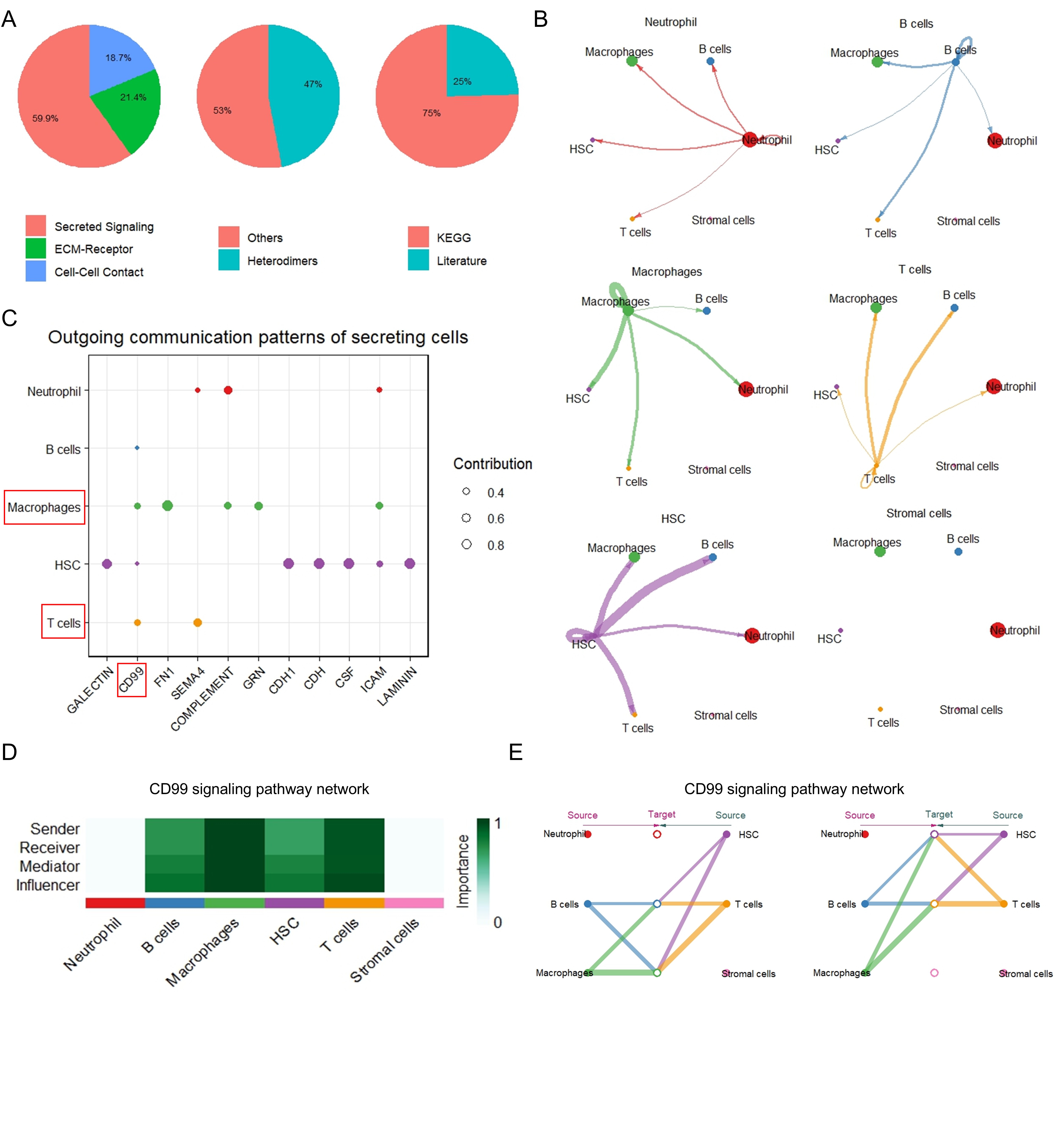
**

**Fig. S3** Crosstalk analysis of macrophages surrounding Ti implants. (**A**) Proportions of autocrine/paracrine signaling interactions, extracellular matrix (ECM)-receptor interactions, and cell-cell contact interactions in the CellChat interaction database. (**B**) Crosstalk between different cell types surrounding Ti implants. (**C**) Contribution of different signals in the outgoing communication patterns of secreting cells. (**D** and **E**) Signal transmission analysis within the *Cd99* signaling network.


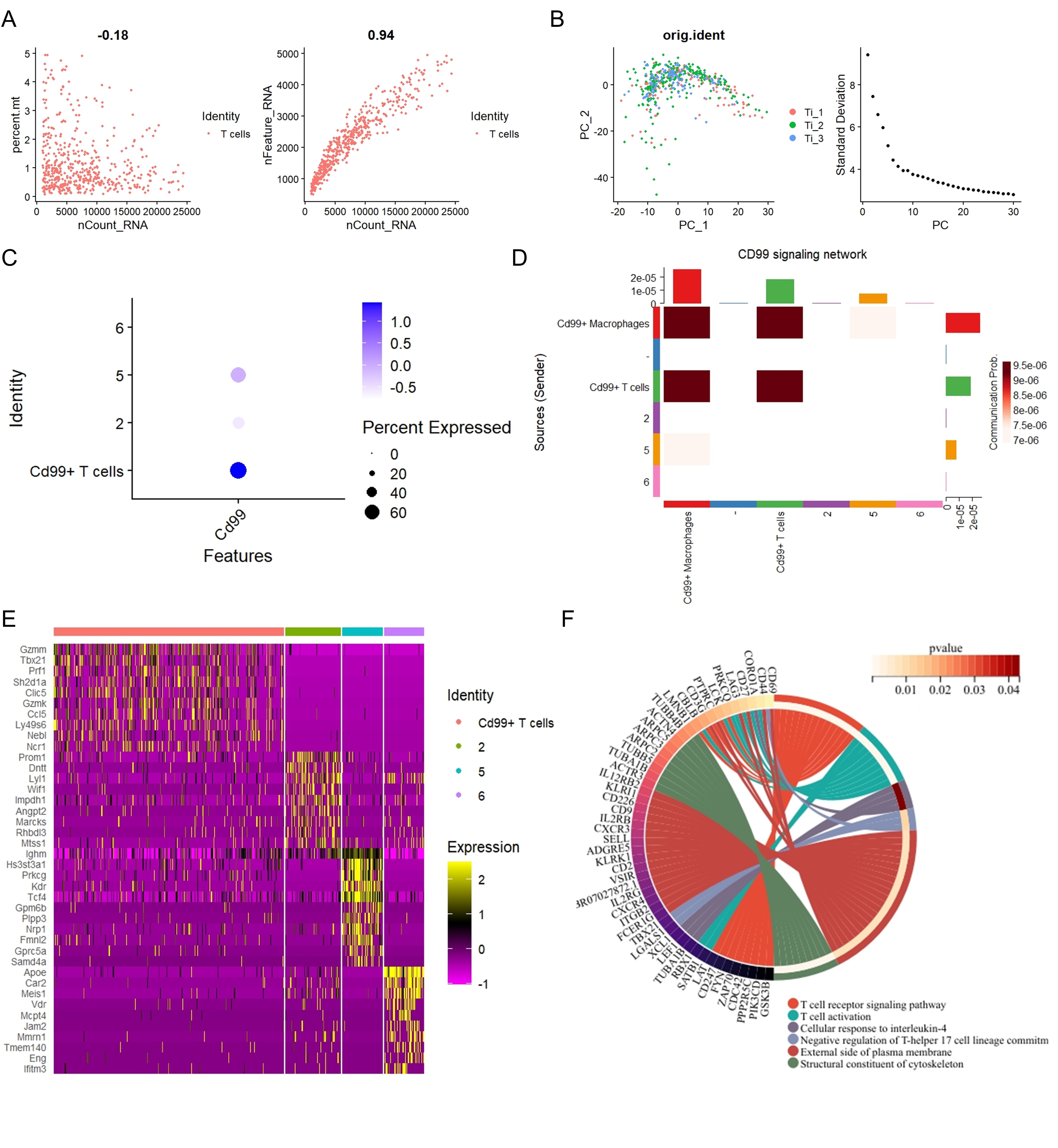


### Fig. S4 Identification and functional analysis of *Cd99*^+^ T cell subpopulations. (A and B) Gene-sample association analysis and PCA-based dimensionality reduction of T cells. (C) Labeling and extraction of *Cd99*^+^ T cells. (D) Heatmap of interaction strength between macrophages and T cells within the CD99 signaling network. (E) Identification and extraction of differentially expressed genes (DEGs) in *Cd99*^+^ T cells. (F) Functional signaling pathways and associated genes in *Cd99*^+^ T cells.


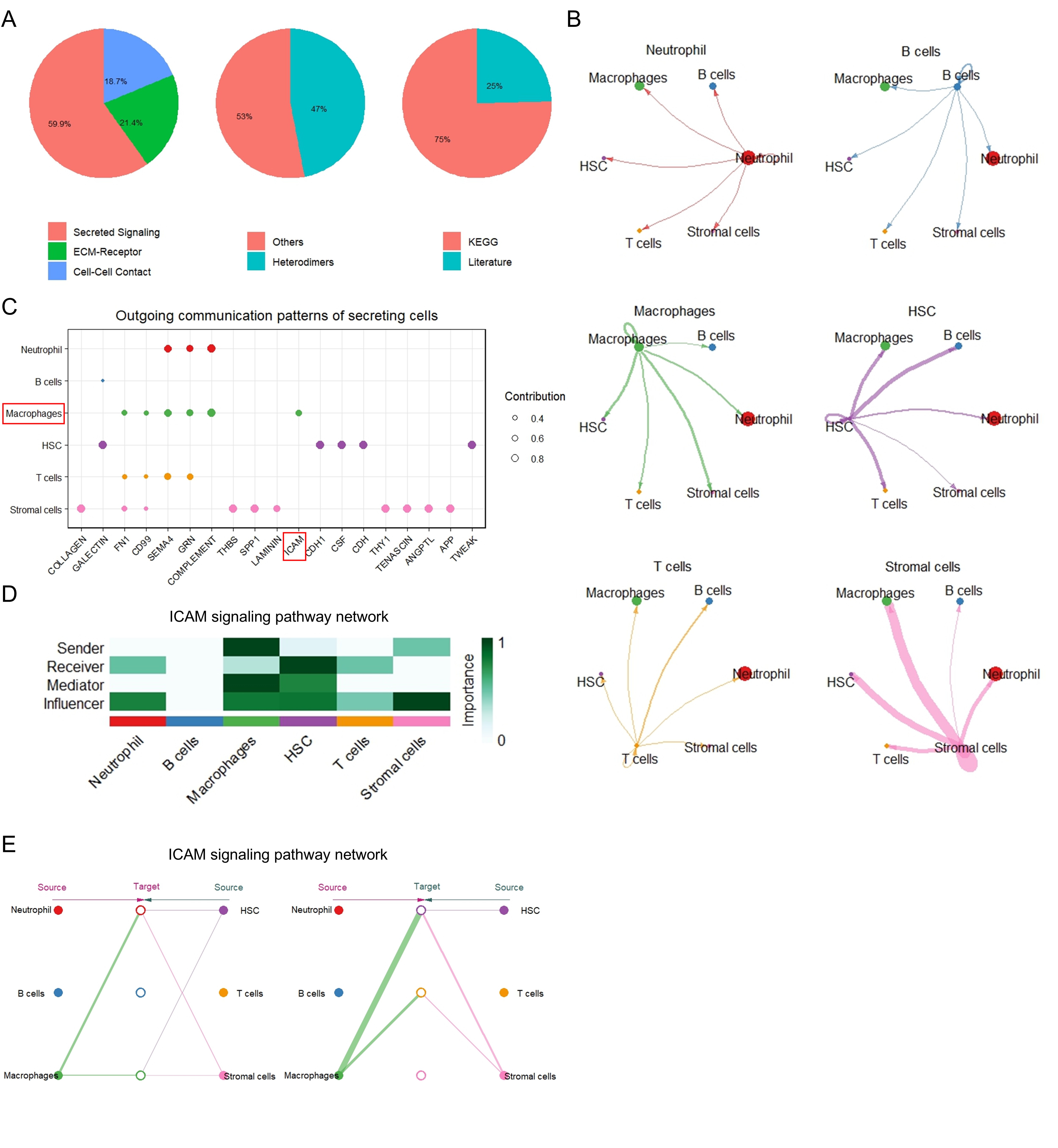


### Fig. S5 Crosstalk analysis of macrophages surrounding PEKK implants. (A) Proportions of autocrine/paracrine signaling interactions, extracellular matrix (ECM)-receptor interactions, and cell-cell contact interactions in the CellChat interaction database. (B) Crosstalk between different cell types surrounding PEKK implants. (C) Contribution of different signals in the outgoing communication patterns of secreting cells. (D and E) Signal transmission analysis within the *Icam1* signaling network.


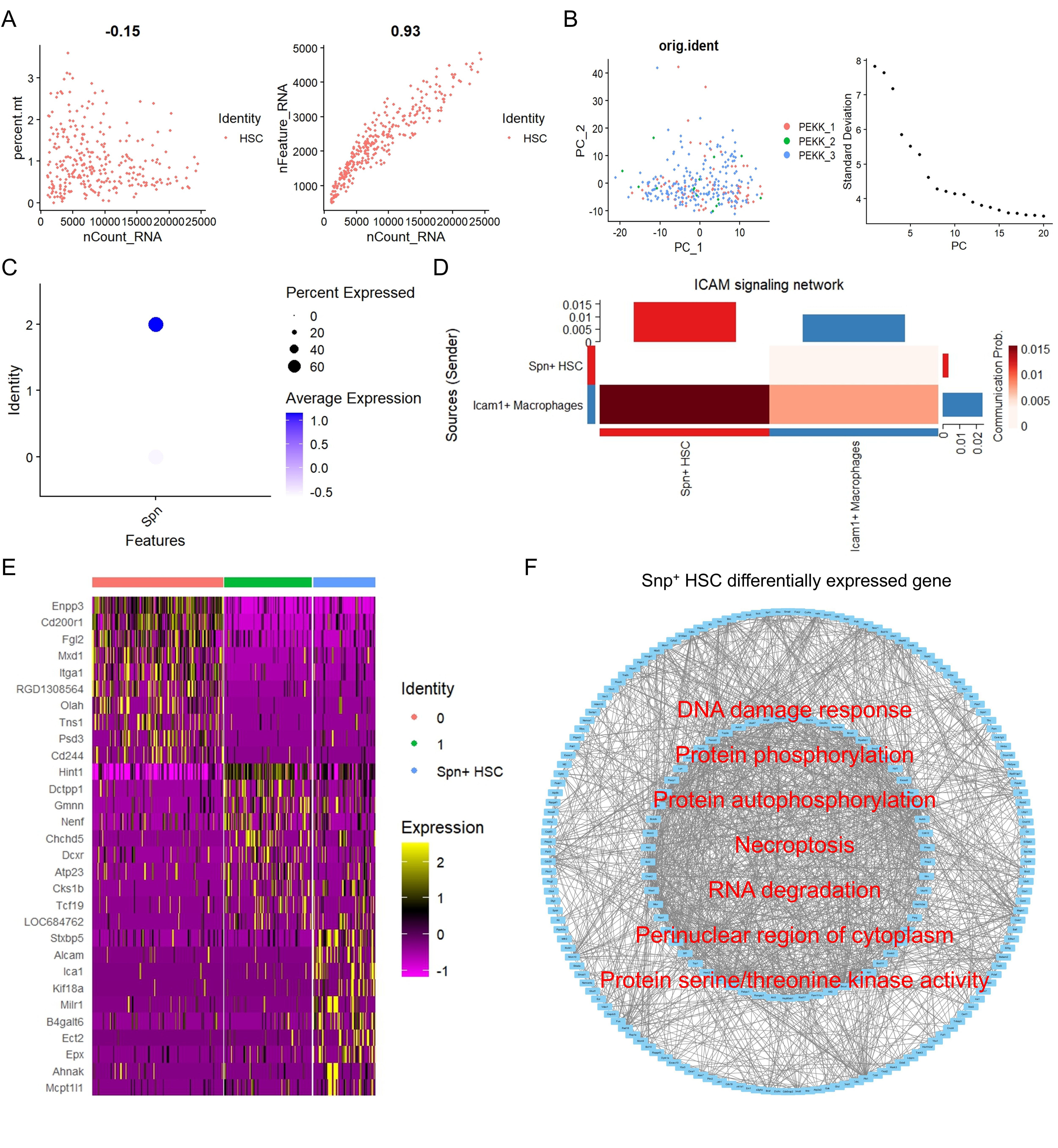


### Fig. S6 Identification and functional analysis of *Spn*^+^ HSCs subpopulations. (A and B) Gene-sample association analysis and PCA-based dimensionality reduction of HSCs. (C) Labeling and extraction of *Spn*^+^ HSCs. (D) Heatmap of interaction strength between macrophages and HSCs within the ICAM signaling network. (E) Identification and extraction of DEGs in *Spn*^+^ HSCs. (F) Functional signaling pathways and associated genes in *Spn*^+^ HSCs.

**References**

[1] S. Jin, C.F. Guerrero-Juarez, L. Zhang, I. Chang, R. Ramos, C.H. Kuan, P. Myung, M.V. Plikus, Q. Nie, Inference and analysis of cell-cell communication using CellChat, Nature communications 12(1) (2021) 1088.

[2] S. Jin, M.V. Plikus, Q. Nie, CellChat for systematic analysis of cell-cell communication from single-cell transcriptomics, Nature protocols 20(1) (2025) 180-219.

[3] G. Dennis, Jr., B.T. Sherman, D.A. Hosack, J. Yang, W. Gao, H.C. Lane, R.A. Lempicki, DAVID: Database for Annotation, Visualization, and Integrated Discovery, Genome biology 4(5) (2003) P3.

[4] M. Kanehisa, M. Furumichi, Y. Sato, M. Kawashima, M. Ishiguro-Watanabe, KEGG for taxonomy-based analysis of pathways and genomes, Nucleic acids research 51(D1) (2023) D587-d592.

[5] T.G.O. Consortium, The Gene Ontology Resource: 20 years and still GOing strong, Nucleic acids research 47(D1) (2019) D330-d338.

[6] X. Qiu, Q. Mao, Y. Tang, L. Wang, R. Chawla, H.A. Pliner, C. Trapnell, Reversed graph embedding resolves complex single-cell trajectories, Nature methods 14(10) (2017) 979-982.

[7] C. Trapnell, D. Cacchiarelli, J. Grimsby, P. Pokharel, S. Li, M. Morse, N.J. Lennon, K.J. Livak, T.S. Mikkelsen, J.L. Rinn, The dynamics and regulators of cell fate decisions are revealed by pseudotemporal ordering of single cells, Nature biotechnology 32(4) (2014) 381-386.
